# IBD-Specific Cognitive Behavioral Therapy: Sustainability of Effect After Three Years

**DOI:** 10.32872/cpe.14717

**Published:** 2025-08-29

**Authors:** Floor Bennebroek Evertsz’, Florentine L. S. Goes, Pieter C. F. Stokkers, Robbert Sanderman, Mathilde G. E. Verdam, Mirjam Sprangers, Claudi L. Bockting

**Affiliations:** 1 Department of Medical Psychology, Amsterdam University Medical Centers, Location AMC, University of Amsterdam, Amsterdam, The Netherlands; 2 GGZ NHN, Amsterdam, The Netherlands; 3 Department of Gastroenterology and Hepatology, OLVG West, Amsterdam, The Netherlands; 4 Department of Health Psychology, University Medical Center Groningen, Groningen, The Netherlands; 5 Amsterdam Public Health Research Institute, Mental Health, Amsterdam, The Netherlands; 6 Department of Psychiatry, Amsterdam University Medical Centers, Location AMC, Amsterdam Public Health, University of Amsterdam, Amsterdam, The Netherlands; 7 Centre for Urban Mental Health, University of Amsterdam, Amsterdam, The Netherlands; Philipps-University of Marburg, Marburg, Germany

**Keywords:** Cognitive Behavioral Therapy, Inflammatory Bowel Disease, three year follow-up, quality of life, anxiety, depression

## Abstract

**Background and Aims:**

‘Inflammatory Bowel Disease (IBD)-specific-Cognitive-Behavioral Therapy’ (CBT) is effective in improving Quality of Life (QoL) and in decreasing anxiety and depression in IBD-patients with poor mental QoL, one month after completing CBT. Main aim was to examine the sustainability of treatment effects up to three years after treatment with CBT.

**Method:**

Participants (*n* = 118) of a previously conducted randomized-control-study on the effects of ‘IBD-specific-CBT’ for IBD-patients were contacted for a long-term follow-up assessment on main outcomes: generic and IBD-specific-QoL (SF-36, IBDQ), anxiety and depression (HADS, CES-D) and DSM-IV disorders (SCID-I). Change over time was examined with multilevel-regression-analyses.

**Results:**

Three years after finishing ‘IBD-specific-CBT’, 61 IBD-patients (response rate 52%) completed the follow-up SCID-I assessment and 52 patients (response rate 44%) completed the assessments for symptomatology. There were no differences between dropouts and participants at three year follow-up, except for a longer disease duration in dropouts. At three-year follow-up the chance of patients having a DSM-disorder significantly decreased with an estimated 48% (from 87% at baseline to 38% at follow-up). Multilevel analyses showed a significant improvement between baseline (*n* = 118) and follow-up measurements (*n* = 52) on outcomes: IBDQ-Total (Cohen’s *d* effect-size *=* .89), SF-36 Physical (*d* = .54), and SF-36 Mental (*d =* .69), HADS-A (*d = -.*77), HADS-D (*d = -*.65) and CES-D (*d = -.*55); all *p* < .01. QoL outcomes showed further improvement between completion (*n* = 90 for IBD-specific QoL and *n* = 91 for generic QoL) and follow-up measurements, with significant improvements for IBDQ-Total (*d* = 0.31) and SF-36 Physical (*d* = 0.32).

**Conclusions:**

Sustainable positive effects of ‘IBD-specific-CBT’ for IBD-patients with poor mental QoL were found and the prevalence of mental conditions substantially decreased over three year follow-up.

## Highlights

- IBD-specific-CBT showed lasting benefits on QoL, anxiety, and depression three years post-treatment.
- Patients further improved, even after completion of therapy regarding IBD-specific QoL and generic physical QoL.
- Comorbid psychiatric disorders at baseline had decreased at long-term follow-up three years after ending ‘IBD-specific-CBT’.
- Larger studies are needed to confirm these findings.

The unpredictability of the chronic relapsing and remitting inflammatory bowel diseases (IBD) ulcerative colitis (UC) and Crohn’s disease (CD), may impact psychological well-being profoundly. Whereas the exact mechanisms by which IBD is developed remains unclear, it is assumed that the disease develops due to an interaction of genetic predispositions, enviromental and dietary factors, gut microbiome and a dysregulated immune response (Lee & Chang, 2021).

Patients with IBD report a poor quality of life (QoL) and psychiatric complaints such as anxiety and depressive disorders (Bennebroek Evertsz’, Thijssens, et al., 2012). Moreover, a recent systematic review and meta-analysis demonstrated a high prevalence of symptoms of common mental health disorders in patients with IBD. According to validated screening instruments, one-third of IBD-patients are affected by symptoms of anxiety and a quarter by symptoms of depression in the previous 10-15 years (Barberio et al., 2021). Moreover, patients with active IBD were also found to have a higher probability of experiencing both anxiety and depression symptoms compared to patients with inactive IBD (Barberio et al., 2021). Although the causal pathway remains unclear, associations between depression, anxiety and disease activity are well demonstrated (Mikocka-Walus et al., 2016). Additionally, anxiety and depression were found to exacerbate disease outcomes in IBD patients (i.e. higher rates of surgery, hospitalization and corticosteroid use) (Keefer, 2021). Moreover, anxiety related irrational beliefs may lead IBD patients to disengage from regular restrictive physical activity (Gravina et al., 2023) and anxiety itself may reduce their therapeutic adherence, as was documented during the COVID pandemic (Pellegrino et al., 2022). Various studies point to a bidirectional link between the brain-gut axis (Gracie et al., 2018). This bidirectional link has been proven to affect the natural course of the disease along with mental health (Fairbrass et al., 2022; Peppas et al., 2021). Furthermore, individuals with a history of depression may show an increased risk of developing subsequent new-onset IBD (Piovani et al., 2024). A recent study found that IBD patients with anxiety and depressive symptoms have a higher risk of developing steroid resistance and IBD related poor outcomes (Duan et al., 2023). Additionally, two recent systematic reviews and meta-analyses (Naude et al., 2023; Seaton et al., 2024) have shown that psychological treatments, including CBT addressing mood disorders, also improve inflammatory biomarkers in IBD (i.e. faecal calprotectin and C-Reactive Protein). Consequently, an effective psychological intervention to target both anxiety and depression in IBD patients is important. Hence, we designed an ‘IBD-specific cognitive behavioral therapy (CBT)’ (Bennebroek Evertsz’, Bockting, et al., 2012), that targets anxiety, depression, Post Traumatic Stress Disorder (PTSD) and adjustment disorders for IBD patients with poor mental QoL. The beneficial effects were measured one month after ‘IBD-specific-CBT’ treatment as studied in a multi-center randomized control trial (QL!C study) (Bennebroek Evertsz’ et al., 2017). The QL!C-study consisted of an experimental group receiving immediate CBT (*n* = 59), and was compared with a wait-list control group receiving standard medical care followed by CBT (*n* = 59) (Bennebroek Evertsz’ et al., 2017). At baseline, (before starting the ‘IBD-specific-CBT’) we measured the prevalence of psychiatric disorders (SCID I), generic and disease specific QoL (SF-36, IBDQ), anxiety and depression (HADS, CES-D) in this IBD-patient group with poor mental QoL (Bennebroek Evertsz’ et al., 2017, 2020). We found a positive effect of ‘IBD-specific-CBT’ on disease specific QoL, generic QoL, depression and anxiety symptoms, one month after completion. Currently, the evidenced based ‘IBD-specific-CBT’ intervention for IBD patients has been implemented and disseminated in several hospitals in the Netherlands and showed comparable positive short–term effects on IBD-specific QoL, anxiety and depression (Bennebroek Evertsz’ et al., 2024).

## Study Aims

The primary aim was to investigate whether treatment gains of ‘IBD-specific-CBT’ (Bennebroek Evertsz’ et al., 2017) were sustained up to three years after treatment with CBT, particularly with respect to psychiatric disorders, disease specific QoL, generic quality of life, anxiety and depressive symptoms.

## Materials and Method

### Procedures

Three years after completion of the ‘IBD-specific-CBT’ from the QL!C-study (Bennebroek Evertsz’ et al., 2017), IBD-patients ‘who participated in the QLIC-study’ were asked to participate in a follow-up study during the period July 2013 and June 2015.

Before participating in the previous QL!C study (Bennebroek Evertsz’ et al., 2017), all the participants had provided written informed consent, including permission to be approached for follow-up research measuring the long-term effect of ‘IBD-specific-CBT’. The patients met the criteria of the diagnoses of Crohn’s disease or ulcerative colitis at the start of the original study. IBD was diagnosed based on the usual clinical criteria, comprising clinical history, physical examination, laboratory findings, negative stool cultures, radiological imaging and endoscopic and histological examinations as assessed by IBD-experts, at least 3-6 months before entry in the QL!C-study (Maaser et al., 2019). Potential participants of the former QL!C study (*n* = 118) were contacted by telephone by a research assistant. If they were interested in receiving more information, we sent them a letter with information about the follow-up study and an invitation for participation. The invitation letter was sent without an initial phone call, if only a postal e-mail address was known. Once patients agreed to participate, an appointment was made for a telephone interview to administer the SCID-I. The questionnaires were sent by mail or e-mail (with a link to the online version of the questionnaires). A reminder was sent if the participant did not return the completed questionnaires by post or e-mail within two weeks. When participants declined participation, they were asked for their permission by phone to fill in the declaration form with the reasons for refusal, demographic- and clinical characteristics.

The QL!C study was approved by the local Medical Ethics Committee of the Amsterdam University Medical Centre (location AMC: dossier number: MEC 08/295) and the long term follow-up study was approved as an amendment of the QL!C study (location AMC: dossier number: MEC NL22948.018.08).

### Outcomes

Psychiatric disorders were assessed with the Structural Clinical Interview for DSM-IV Disorders (SCID-I) (First et al., 1999), by psychologists who received a specific training.

All other assessments were based on self-report questionnaires, which are the same as the questionnaires used in the original trial (QL!C-study) (Bennebroek Evertsz’ et al., 2017). The primary outcome was the total score on the Inflammatory Bowel Disease Questionnaire (IBDQ) – 32 items assessing four domains; bowel symptoms, systemic symptoms, and emotional and social functioning (Russel et al., 1997). Where higher scores indicate a better health related QoL. Secondary outcome was generic QoL assessed with the SF-36 (Ware, 1992). The SF-36 items can be aggregated into a Physical-Component-Summary (PCS) score and a Mental-Component-Summary (MCS) score. With higher scores indicating better health related QoL. Tertiary outcomes were depression and anxiety, which were assessed using the Hospital Anxiety and Depression Scale (HADS) (Zigmond & Snaith, 1983). The HADS is considered to be unbiased by the presence of a somatic illness. Its 14 items were combined to form an anxiety (7 items) and depression scale (7 items), without including physical symptoms. With higher scores indicating more symptoms. The Depression Scale (CES-D) consisting of 20 items, assesses depressive symptomatology in the general population was also used to examine the difference between these two questionnaires on depression (Radloff, 1977).

### Statistical Analysis

Data analyses were performed using IBM SPSS (version 28.0.1.1 (15)). The threshold for significance was *p* < .05 (two tailed). Demographic and clinical characteristics were analyzed using frequencies, percentages and median scores. Baseline differences between the group responders and non-responders were compared with Chi-square or Wilcoxon tests. Normality assumptions were checked using the Shapiro-Wilk test and inspection of histograms, appropriate non-parametric tests (i.e. Wilcoxon tests) were applied if needed. Scores on disease specific and generic QoL, anxiety and depression symptoms at baseline (before starting the IBD-specific-CBT) were compared with scores one month after completion and three years after CBT. Additionally, the prevalence of mental condition and co-morbid mental conditions at baseline and three years after CBT were compared. To analyze differences over time we used multilevel regression analyses (Heck & Thomas, 2020). Multilevel modeling takes into account the relationship between measures of the same individual across time and allows for including all available data (i.e. also data from participants who did not complete all measurements). Multilevel logistic regression was used to assess the difference between psychiatric disorders (DSM-IV) as assessed at baseline and the three-year follow-up measurements. The analysis yields estimated probability of having a psychiatric disorder at both measurements, where the odds-ratio (*OR*) is used to assess the difference between these two probabilities. When the upper bound of the 95% confidence interval of the estimated odds-ratio is below “1” this indicates that the chance of having a psychiatric disorder has significantly decreased with *p* < .05. Differences between scores on baseline, completion (post-CBT) and three-year follow-up measurements of IBD-specific and generic QoL, anxiety and depression symptoms were assessed with multilevel regression analysis. Comparison between baseline and three-year follow-up was used to measure change over time, and between CBT completion and follow-up was used to assess whether the treatment effectiveness was sustained after three years. This analysis yields estimated mean differences between assessments, where confidence intervals of the estimated effects can be used to assess statistical significance of the difference. When the 95% confidence interval does not include zero, this indicates that the estimated difference is statistically significant at alpha = .05. The estimated effects can also be used to calculate a Cohen’s *d* effect size (ES with 0.3, 0.5 and 0.8 indicating a small, moderate and large effect, respectively) while taking into account the correlation between measurements using the design-effect (Hox et al., 2018). The multilevel analyses were performed using the package lme4 (v1.1-34) (Bates et al., 2015) in R (R Core Team, 2021). Assumptions of homogeneity of variances and normality of residuals were assessed using an Anova on squared residuals within participants and by inspecting QQ-plots respectively. Assumption of (log)linearity does not apply as the comparisons involves only two measurement occasions. We added an ad-hoc analyses to measure the effect of gender, age and disease type.

## Results

Of the 57 (48%) IBD-patients who did not participate in this follow-up study (dropouts), 42 (74%) refused participation with no reason, 9 (16%) did not respond to letters and/or telephone calls, 2 (3%) persons indicated to be physically not able to participate and 4 (7%) persons were not able to participate due to time constraints. The dropout analyses (comparing baseline measures of the 61 patients participating at three-year follow-up vs the 57 dropouts) showed no significant differences regarding demographic and clinical characteristics and psychiatric disorders (DSM-IV) (data not shown). Dropouts had a significantly longer median disease duration of 11 years at time of baseline while participants who participated at follow-up’ had a ‘median’ disease duration of 5 years (*p* = .03).

The demographic- and clinical characteristics of 61 IBD-patients (response rate 52%) who completed the three year follow-up SCID-I telephonic interview assessment, are summarized in Table 1. Median age at long term follow-up was 36 years (IQR 28-47), 35 patients are female (57.4%) and almost more than two-fifth of our participants are married or living together (44.3%). Of the 61 participating IBD-patients there were 52 (85%) IBD-patients who also completed IBD-specific and generic QoL, anxiety and depression symptoms at three-year long-term follow-up. Median age was 35 years (IQR 28-47), 30 patients were female (57.7%) and nearly half of them were married or living together (43.4%) (see also Table 1). There were also no significant differences regarding demographic and clinical characteristics (and neither disease duration; *p* = .07) between the *n* = 52 participants who completed follow-up and patients lost to follow-up (data not shown). Assumptions of homogeneity of variances and normality of residuals were met for all multilevel analyses.

**Table 1:** Demographic and Clinical Characteristics

Variable	IBD (*n* = 61) Baseline and 3 year follow-up	IBD (*n* = 52) Baseline, post-CBT and 3 year follow-up
	Completion of Psychiatric Disorders	Completion of IBDQ, SF-36, HADS, CES-D
Gender		
Female	35 (57.4%)	30 (57.7%)
**Age in years** (Median [IQR])	36 (28-47)	35 (28-47)
Marital status		
In a relationship	27 (44.3%)	23 (43.4%)
Level of education		
Low (Primary or Secondary)	24 (39.4%)	23 (40.4%)
High (College or University)	32 (52.5%)	26 (50%)
Otherwise	5 (8.2%)	3 (5.7%)
Employment		
Employed or studying	34 (55.7%)	28 (53.8%)
Unemployed	27 (44.3%)	24 (46.2%)
**Sickleave**	12 (19.7%)	10 (19.2%)
Hospital type		
Academic	36 (59%)	29 (55.8%)
Diagnosis		
Ulcerative colitis	26 (42.6%)	22 (42.3%)
Crohn’s disease	35 (57.4%)	30 (57.7%)
**Disease duration in years** (Median IQR)	5 (3 – 13)	6 (3 – 13)
Number of operations		
None	43 (70.5%)	36 (69.2%)
≥1	18 (29.5%)	16 (30.8%)
**Stoma**	1 (1.6%)	0 (0%)
Medication with side-effect depression		
Prednisone	12 (19.7%)	9 (17.3%)
None	49 (80.3%)	43 (82.7%)

Multilevel regression analyses showed a significant decrease in estimated probability of overall psychiatric disorders (baseline 86.7% (*n* = 51), follow-up 38.3% (*n* = 24), *p* < .01), mood disorders (baseline 45.4% (*n* = 25), follow-up 20% (*n* = 13), *p* < .01), anxiety disorders (baseline 35.1% (*n* = 21), follow-up 10.7% (*n* = 8), *p* < .01) and adjustment disorders (baseline 30.6% (*n* = 23), follow-up 11.5% (*n* = 7), *p* < .01) (see Table 2). For the other psychiatric disorders, including eating disorder, alcohol related disorder and psychotic disorder, the number of participants with the disorder at baseline was very low (*n* < 5), precluding further analyses.

**Table 2:** Frequencies of Psychiatric Disorders of IBD Patients at Baseline and at 3 Year Follow-Up

Psychiatric disorder	Baseline (*n* = 118)		3 year follow-up (*n* = 61)		*OR* [95% CI]
	*n*	%^a^	*n*	%^a^	
DSM-IV	51	86.7	24	38.3	0.10 [0.02, 0.23]**
Mood	25	45.4	13	20.0	0.30 [0.13, 0.62]**
Anxiety	21	35.1	8	10.7	0.22 [0.07, 0.52]**
Adjustment	23	30.6	7	11.5	0.30 [0.11, 0.68]**

*Note.* Psychiatric disorders were only assessed at baseline and at 3 year follow-up and are not available immediately at post-CBT.

^a^Percentages are based on the estimated odds from the multilevel logistic regression model.

**p < .01.

Additionaly, analyses showed a significant improvement in IBD-specific QoL between baseline and follow-up among 52 patients after three years (IBDQ total score: Cohen’s *d* = .89, IBDQ bowel: Cohen’s *d* = .69, IBDQ systemic: Cohen’s *d* = .79, IBDQ emotional: Cohen’s *d* = .79, IBDQ social: Cohen’s *d* = 73). Also there was a significant positive long-term effect on generic QoL (SF-36 physical: Cohen’s *d =* .54 and SF-36 mental: Cohen’s *d* = .69). Strikingly, patients showed significantly further improvement three years after completion of ‘IBD-specific-CBT’, concerning IBD-specific QoL total, IBDQ bowel, IBDQ social and generic physical QoL (completion versus follow-up IBDQ total score: Cohen’s *d =* .31; IBDQ bowel: Cohen’s *d* = .34, IBDQ social: Cohen’s *d* = .33, SF-36 physical: Cohen’s *d =* .32) (see Table 3).

**Table 3:** Comparison of QoL, Anxiety and Depression Between Baseline (n = 103) and 3 year Follow-Up (n = 52), and Completion (Post-CBT; n = 91 (IBDQ) or n = 90 (Other PROMs)) and Follow-Up Assessments (Total n = 106)

Assessment	Baseline vs Follow-up		Completion (post CBT) vs Follow-up	
	MD^a^ [95% CI]	Cohen’s *d*^b^	MD^a^ [95% CI]	Cohen’s *d*^b^
IBDQ Total	25.11 [17.92, 32.31]**	0.89	8.85 [1.60, 16.05]**	0.31
IBDQ Bowel	6.8 [4.26, 9.33]**	0.69	3.41 [0.86, 5.95]**	0.34
IBDQ Systemic	4.53 [3.00, 6.06]**	0.79	1.34 [-0.19, 2.87]	0.23
IBDQ Emotional	10.1 [7.01, 13.19]**	0.79	2.32 [-0.79, 5.42]	0.18
IBDQ Social	3.72 [2.37, 5.07]**	0.73	1.67 [0.32, 3.02]**	0.33
SF-36 Physical	2.07 [1.01, 3.14]**	0.54	1.22 [0.16, 2.28]*	0.32
SF-36 Mental	3.75 [2.49, 5.03]**	0.69	0.8 [-0.48, 2.08]	0.15
HADS Anxiety	-3.23 [-4.29, -2.18]**	-0.77	-0.85 [-1.90, 0.21]	-0.20
HADS Depression	-2.39 [-3.39, -1.39]**	-0.65	-0.67 [-1.66, 0.33]	-0.18
CES-D	-3.83 [-5.52, -2.14]**	-0.55	-1.56 [-3.26, 0.15]	-0.22

*Note.* Cohen’s *d* .3 is small, .5 is medium and .8 is large. IBDQ = Inflammatory Bowel Disease Questionnaire; SF-36 = MOS 36-item Short Form Health Survey; HADS = Hospital Anxiety and Depression Scale; CES-D = The Center for Epidemiologic Studies Depression Scale.

^a^Mean difference (MD) is based on the estimated effect in the multilevel regression model. ^b^Cohen's *d* is based on the estimated parameters from the multilevel regression model.

*p < .05. **p < .01.

Anxiety and depression symptoms remained decreased three years after completion of ‘IBD-specific-CBT’ (baseline versus follow-up HADS-A: *p* < .01, Cohen’s *d = -*.59; HADS-D: *p* < .01, Cohen’s *d = -*.41 and CES-D: *p* < .01, Cohen’s *d = -*.66). However, these results did not further significantly improve after completion of the therapy (completion versus follow-up HADS-A: *p* = .42, Cohen’s *d = -*.12; HADS-D: *p* = .67, Cohen’s *d = -*.06; CES-D: *p* = .20, Cohen’s *d = -*.19).

Ad hoc explorative analyses showed that there were some effects of disease-type (i.e., UC versus CD). Participants with CU showed a larger improvement between baseline and three year follow up on IBDQ total (*p* < .05), and the improvement between completion and three-year follow up for SF36-MH was only statistically significant for participants with CU (but not for participants with CD). There were no effects of age and gender. There were also no effects of gender, age and disease-type on the decrease of psychiatric disorders over time.

## Discussion

We found a sustainable positive effect up to 3 years follow-up of ‘IBD-specific-CBT’ on IBD-specific and generic QoL, anxiety- and depressive symptoms among IBD patients with a priori poor mental QoL. Moreover, comorbid psychiatric disorders at baseline had decreased at long-term follow-up three years after ending ‘IBD-specific-CBT’.

Recent meta-analyses and reviews did find short term effectiveness of CBT for IBD on mental health problems (such as anxiety and depression) (Chen et al., 2021; Li et al., 2019). However, to date, positive sustainable effects of ‘IBD-specific-CBT’ have not been demonstrated.

After 24 months of observation, Mikocka-Walus et al. (2017) did not find a significant longterm effect of CBT on QoL, mental health or coping. A study of McCombie et al. (2016), that examined the long-term effect of computerized CBT, found an increased QoL at 12 weeks but the effect was not maintained after 6 months. In patients with other medical illnesses, little is known about the sustainable effects on mental symptomatology and mental condition as well as QoL after CBT (van Straten et al., 2010). However, sustainable effects of CBT have been reported for depressive disorder (Cuijpers et al., 2013, 2023; Furukawa et al., 2021; Legemaat et al., 2023).

We would recommend a number of amendments for future research to investigate the sustainable effects of ‘IBD specific CBT’. First, employment of a larger sample size that would accommodate dropout, enabling the study of long-term effects. Furthermore, inclusion of multiple assessment points (e.g., 3, 6 and 12 months after completion) would enable a closer examination of the treatment effects over time. Even more so, in the first phase of this study we randomized CBT to a wait-list control group. Therefore we have no control group for the three year follow-up. We would recommend to use a different type of control-group. Namely comparison of ‘IBD-specific-CBT’ with IBD patients receiving only treatment as usual for longterm follow-ups. Fortunately, this research will be repeated with due observance of these recommendations.

Given these promising short-term and long-term results of ‘IBD-specific-CBT’ in IBD patients with poor mental QoL and the positive results of the aforementioned implementation study of ‘IBD-specific-CBT’ in four hospitals in the Netherlands (Bennebroek Evertsz’ et al., 2024), there is an urgent need to disseminate standard stepwise screening and treatment of mental health disorders in IBD patients. As mentioned before CBT for patients living with IBD not only enhances psychological benefit, but may also improve their physical health (e.g. inflammation) (Naude et al., 2023; Seaton et al., 2024).

Moreover, providing integrated psychological care to IBD patients in need for mental health can reduce costs, particularly by decreasing visits to emergency departments (Lores et al., 2021). However, untill now the integration of mental health care in general IBD care is still insufficient and a challenge in daily clinical practice (Fairbrass & Gracie, 2021; Peppas et al., 2021).

### Conclusions

IBD patients with initial poor mental QoL were found to experience a sustainable positive effect of the ‘IBD-specific-CBT’ on IBD-specific and generic QoL, anxiety and depressive symptoms, three years after ending CBT. Morover, a significant reduction of the prevalence of mental conditions was found (83.6% 1 month after CBT to 39.3% with a mental health condition at three year follow-up). The prophylactic effect of CBT seems also to hold for IBD-specific problems, since patients further improved, even after completion of therapy regarding IBD-specific QoL and generic physical QoL. This gives an indication that this therapy may not only have sustainable positive effects, but also generate further improvement over years after completion. This is in line with other findings on Preventive Cognitive Therapy in depression (Bockting et al., 2018; Furukawa et al., 2021; Legemaat et al., 2023) and CBT in anxiety disorders (van Dis et al., 2020).

We can therefore conclude this ‘IBD-specific-CBT’ was found to have sustainable effects in decreasing mental health problems and mental conditions in IBD-patients on the long term. Moreover this study shows the first indications of long-term sustainable effects. Larger studies are needed to confirm these findings.

## Supplementary Materials

The Supplementary Materials contain the preregistration for the study (see Bennebroek Evertsz’, 2025S).



Bennebroek Evertsz’F.
 (2009). Enhancing the quality of life of patients with inflammatory bowel disease: A multi-center study investigating cognitive behavioral therapy
[Preregistration; Trial registration number = 1869]. PsychOpen. https://www.onderzoekmetmensen.nl/nl/trial/19981
10.1186/1471-244X-12-227PMC357039723237076

## Data Availability

The corresponding author FBE had full access to the study data and material. The data underlying this article will be shared on request to the corresponding author. Code availability: Non applicable.

## Reporting Guidelines

Clinical trial, intervention study following the CONSORT statement.

## Preregistration

Before start of the study the trial was registered at the Overview of Medical Research in the Netherlands (OMON), the formerly Dutch Trial Register. Trial registration number of the original randomized control trial (TC = 1869) (see Bennebroek Evertsz’, 2025S).
